# TGF-β1 Improves Nerve Regeneration and Functional Recovery After Sciatic Nerve Injury by Alleviating Inflammation

**DOI:** 10.3390/biomedicines13040872

**Published:** 2025-04-03

**Authors:** Maorong Jiang, Zihan Ding, Yuxiao Huang, Taoran Jiang, Yiming Xia, Dandan Gu, Xi Gu, Huiyuan Bai, Dengbing Yao

**Affiliations:** 1 School of Life Sciences, Key Laboratory of Neuroregeneration of Jiangsu and Ministry of Education, Co-Innovation Center of Neuroregeneration, Nantong University, No. 9 Seyuan Road, Nantong 226019, China; jiangmr@ntu.edu.cn (M.J.);; 2 Medical School of Nantong University, Nantong 226001, China

**Keywords:** TGF-β1, peripheral nerve injury, nerve regeneration, neuronal function recovery, inflammatory response

## Abstract

**Background:** Peripheral nerves have a certain regenerative ability, but their repair and regeneration after injury is a complex process, usually involving a large number of genes and proteins. In a previous study, we analyzed the gene expression profile in rats after sciatic nerve injury and found significant changes in transforming growth factor-beta 1 (TGF-β1) expression, suggesting that TGF-β1 may be involved in the process of nerve regeneration after injury. **Methods**: In this study, we first detected the time-course expression and localization of TGF-β1 in dorsal root ganglion (DRG) tissues in a rat sciatic nerve transection model via RT-qPCR. Secondly, we investigated the bioactive roles of TGF-β1 in primary cultured DRG neuron cells through a CCK8 assay, TUNEL assay, and immunofluorescence staining. Thirdly, we explored the neuroprotective roles of TGF-β1 in an in vivo model of sciatic nerve regeneration through morphological observation, behavioral, and electrophysiological tests, and a molecular biological measure. **Results**: We found that TGF-β1 expression was increased after injury and mainly located in the cytoplasm and nuclei of neuron cells in the DRG. TGF-β1 may regulate the viability, apoptosis, and neurite outgrowth of primary DRG neuron cells. In our in vivo model of sciatic nerve regeneration, TGF-β1 improved nerve regeneration and neuronal function recovery after sciatic nerve injury, alleviated the inflammatory response, and relieved neuropathic pain via the TGF-β1/smad2 pathway. **Conclusions**: This study provides an experimental and theoretical basis for using TGF-β1 as a neuroprotective agent after peripheral nerve injury in clinical practice in the future.

## 1. Introduction

In daily life, damage caused by various accidents and neurodegenerative diseases harms the peripheral nervous system (PNS) and, in severe cases, even affects its ability to control the body, resulting in the loss of neurological function [1,2]. Compared with the central nervous system (CNS), in the PNS, axons can be repaired and regenerated in the case of axon damage, as the microenvironment promotes regeneration and the intrinsic growth force of neurons is induced by reprogramming after injury [3,4,5]. However, the actual outcomes of repair or regeneration are not satisfactory. The sciatic nerve is one of the most frequently injured nerves of the lower extremities and has a complex microanatomy. Developing approaches to better repairing and regenerating sciatic nerves and promoting the recovery of damaged nerve function has always been a major challenge in medicine.

The dorsal root ganglion (DRG) is located on the inner side of the intervertebral foramen of the spine, which is responsible for receiving all nerve impulses from the PNS and transmitting impulses to the spinal cord and, finally, to the brain [6]. So, the DRG is considered a bridge connecting the PNS and the CNS [6,7]. Once peripheral nerves are injured, the DRG is stimulated with intrinsic regenerative force to regenerate a large number of axons [8]. However, this process often requires the cooperation of various nutritional factors and signal pathways [9]. Additionally, relying on the injured nerve’s regenerative ability is not enough to repair the nerve well. There are many factors that affect this process, including the damaged nerve itself and its innervation of target organs, as well as the regenerative microenvironment connecting the nerve and targets [10]. Schwann cells (SCs) and macrophages involved in clearing myelin debris are considered key factors, as well as various neurotrophic factors, the extracellular matrix (ECM), and cell adhesion molecules [8,11]. All kinds of neuron-related factors and signal pathways, as well as the genes whose expression changes after peripheral nerve injury, play very important roles in regeneration [12]. Future studies may bring new ideas for how to maintain the vitality and regeneration of DRG neurons.

Transforming growth factor-beta 1 (TGF-β1) belongs to the TGF superfamily and has been widely studied since it was successfully expressed in *E. coli* in the 1980s [13]. It has been found to play a crucial regulatory role in early embryonic development, cell proliferation and differentiation, and the immune response [13,14]. It has also been reported that TGF-β is a key factor in regulating the inflammatory process and the main regulator in the process of acute tissue healing [15]. In addition, TGF-β is also involved in the construction and remodeling of the ECM, along with the epithelial–mesenchymal transition (EMT), and it plays an important regulatory role in the development of a variety of diseases (such as autoimmune diseases, cardiovascular diseases, cancer, and neurodegenerative diseases) [16,17]. Moreover, TGF-β participates in the regulation of SCs and macrophages and the construction of the regeneration microenvironment [18].

In our previous study, gene chip microarrays were applied to analyze gene expression in the early Wallerian degeneration (WD) of the distal nerve stump at 0, 0.5, 1, 6, 12, and 24 h after rat sciatic nerve injury [19]. Kyoto Encyclopedia of Genes and Genomes (KEGG) pathway analysis identified numerous pathways, including TGF-β signaling. Therefore, we proposed that TGF-β signaling plays an important role in early WD. However, the roles and molecular mechanisms of TGF-β1 in peripheral nerve regeneration after injury remained unclear. Thus, in this study, we investigated the time-course expression of TGF-β1 and explored its roles and mechanisms in nerve regeneration. Our findings provide an experimental and theoretical basis for using TGF-β1 for nerve regeneration and functional recovery after peripheral nerve injury in clinical practice in the future.

## 2. Methods

### 2.1. Animals

Male Sprague-Dawley (SD) rats (8 weeks of age, 180–200 g body weight) were obtained from the Laboratory Animal Center of Nantong University and kept in a room with constant temperature and humidity and a light–dark (12:12 h) cycle.

### 2.2. Animal Models

All animal experiments protocols were approved by the Institutional Animal Care and Use Committee of Nantong University (approval No. 2019-nsfc004), and animal models were conducted according to the guide for the Care and Use of Laboratory Animals. All experiments were designed and reported according to the Animal Research: Reporting of In Vivo Experiments (ARRIVE) guidelines [20].

Based on previous reports, the sciatic nerve transection model was established [21]. In brief, after rats were anesthetized with an intraperitoneal injection of complex anesthetics (10 mg/kg xylazine, 95 mg/kg ketamine, 0.7 mg/kg acepromazine), the sciatic nerve was exposed and transected with a 1 cm gap. Additionally, the sciatic nerve regeneration model was performed as follows. A 0.7 cm silicone tube (Invitrogen, New York, NJ, USA) was sutured between the two stump ends, after which the sciatic nerve of the left leg was exposed and transected with a 0.5 cm gap.

### 2.3. Real-Time Quantality PCR (RT-qPCR)

The L4 and L5 DRG tissues from the sciatic nerve transection model and regeneration model, DRG neuron cells after transfection with lentivirus, and proximal tissues of sciatic nerve from the regeneration model were harvested. TRIzol reagent (ThermoFisher, Waltham, MA, USA) was applied to extract total RNA from tissues or cells, and first strand cDNA was synthesized using an RT kit (ThermoFisher, Waltham, MA, USA). qPCR was performed using a real-time PCR system (Applied Biosystems, Foster City, CA, USA). All primers used in this study are listed in Table 1.

### 2.4. Western Blot

Standard procedures for Western blotting analysis were performed as previously described [22]. The L4 and L5 DRG tissues from the sciatic nerve transection model and regeneration model, DRG neuron cells after transfection with lentivirus, and proximal tissues of sciatic nerve from the regeneration model were harvested. The primary antibodies and secondary antibodies used are listed in Table 2. ImageJ software 1.54g (NIH, Bethesda, MD, USA) was applied to quantify the target signal.

### 2.5. Primary DRG Neuron Cell Culture and Treatment

Primary DRG neuron cells were cultured as described previously [23]. Briefly, DRGs were removed from a newborn SD rat (1–3 days old), and digested with 1% collagenase for 30 min and 0.25% trypsin for 20 min at 37 °C. The mixture was triturated, centrifuged, and re-suspended in Neurobasal-A medium (Gibco, Carlsbad, CA, USA). The cell pellets were plated on poly-L-lysine pre-coated plastic culture plates.

The primary DRG neuron cells, following culturing for 24 h, were transfected with lentivirus to knockdown or express TGF-β1. The lentivirus used to knockdown or overexpress TGF-β1 was obtained from Vigen Biotechnology Co., Ltd. (Zhenjiang, China). The volume of virus was calculated with multiplicity of infection (MOI), and 5 μg/mL polybrene was used to improve the transfection rate. The DRG neuron cells were incubated overnight at 37 °C with 5% CO_2_. The knockdown or overexpression was confirmed using the observation of green fluorescence, RT-qPCR, and Western blotting.

### 2.6. CCK8 Assay and TUNEL Staining

The CCK8 kit and TUNEL staining kit were both obtained from Vazyme (Nanjing, China). After the DRG neuron cells were transfected for 48 h, the CCK8 kit or TUNEL kit was applied for detection according to the product manual.

### 2.7. Immunofluorescence (IF) Staining

DRG tissues, the primary cultured DRG neuron cells and transfected neuron cells, were fixed in 4% paraformaldehyde (PFA) and incubated with primary anti-TGF-β1 or anti-Tuj1 at 4 °C overnight. The proximal end tissues of sciatic nerve, at 1, 4, and 7 days after injection with lentivirus, were fixed with 4% PFA and incubated with anti-F4/80 primary antibody. Then, the tissues and neuron cells were further incubated with Cy3-conjugated or Coralite488-conjugated secondary antibodies for 2 h at room temperature. Additionally, these cells were also stained with DAPI at 37 °C for 10 min. The fluorescence signal was observed under a ZEISS Axio Scope.A1 microscope (ZEISS, Oberkochen, Germany). To quantify neurite outgrowth from DRG neuron cells, the neurite length was measured using ImageJ software (NIH, Bethesda, MD, USA) as previously described [24].

The collected DRG tissues and sciatic nerve tissues were stained with primary antibodies and secondary antibodies using the same methods as above. The primary antibodies and secondary antibodies used are listed in Table 2.

### 2.8. Lentivirus Injection

After the rat sciatic nerve regeneration models were established, lentivirus was intrathecally injected to knockdown or overexpress TGF-β1. As previously described [25], in brief, a glass electrode needle connected to the microinjector was inserted obliquely behind the intervertebral foramen, and each rat was injected with 10 μL of lentivirus using a Micro4 microinjection pump.

### 2.9. Hematoxylin and Eosin (H&E) Staining

At 8 weeks after the rats had been injected with lentivirus in the sciatic nerve regeneration model, gastrocnemius muscles both operated and contralateral were collected, after narcotism, and weighed to calculate muscle wet weight ratio. Next, the gastrocnemius muscles were post-fixed in 4% PFA, embedded in paraffin, and cut into 5 μm thick sections. ImageJ software (NIH, Bethesda, MD, USA) was applied to measure the cross-sectional area of the muscle fibers after H&E staining.

### 2.10. Behavioral Tests

The sciatic nerve function index (SFI) analysis and thermal pain analysis were both performed by researchers blinded to group assignments.

### 2.11. SFI Analysis

The SFI analysis was performed using Catwalk gait analysis system (Noldus, Leesburg, VA, USA). In the sciatic nerve regeneration model, the footprints of the rats were recorded using a Catwalk fluorescent plate at 2, 4, 6, and 8 weeks following lentivirus injection. So, the print length (PL), the toe spread (TS), and the intermediary toe spread (IT) of non-operated (N) and experimental (E) hind legs were obtained. The SFI was calculated with the formula [21]: SFI = 109.5 (ETS − NTS)/NTS − 38.3 (EPL − NPL)/NPL + 13.3 (EIT − NIT)/NIT − 8.8.

### 2.12. Thermal Pain Analysis

The Randall–Selitto Assay (IITC Life Sciences, Woodland Hills, CA, USA) was applied to perform thermal pain analysis. At 1, 2, 4, 6, and 8 weeks following lentivirus injection, in the sciatic nerve regeneration model, the time of contraction induced by light stimulation was recorded.

### 2.13. Electrophysiological Test

The rat sciatic nerve was re-exposed after anesthesia, and compound muscle action potentials (CMAPs) were recorded on the gastrocnemius after the sciatic nerve trunk was stimulated using electric current.

### 2.14. Transmission Electron Microscope Analysis

The sciatic nerve tissues were collected, fixed with 2.5% glutaraldehyde, and post-fixed with 1% osmium tetroxide solution. The ultrathin sections were observed under a transmission electron microscope (JEOL Ltd., Tokyo, Japan) after being stained with lead citrate and uranyl acetate.

### 2.15. Statistical Analysis

Data were analyzed using GraphPad Prism software (version 8) (GraphPad, La Jolla, CA, USA). All data were expressed as means ± SEM. One-way ANOVA with subsequent Turkey’s tests was applied to analyze statistical differences between groups. Differences were considered statistically significant at *p* < 0.05.

## 3. Results

### 3.1. Expression of TGF-β1 in DRG After Sciatic Nerve Injury

**Figure 1:**
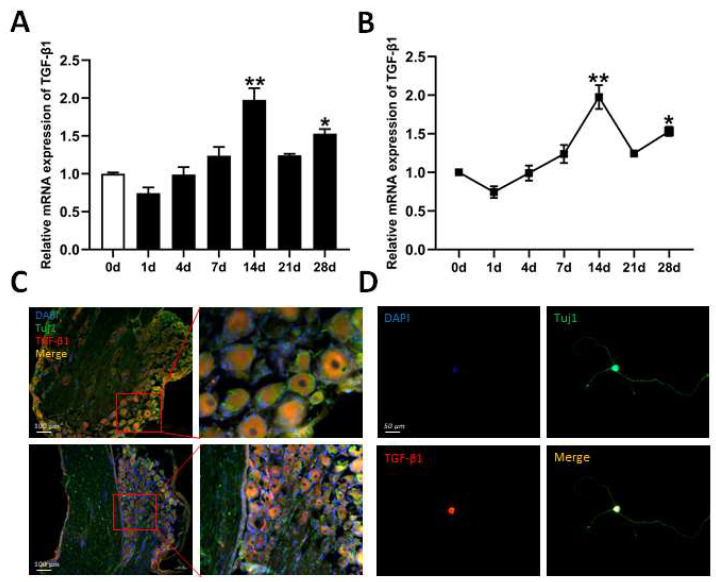
Time-course expression and localization of TGF-β1. (**A**,**B**) Time-course mRNA expression of TGF-β1 in L4 and L5 DRG tissues in rats after sciatic nerve transection via RT-qPCR. Versus od, * *p* < 0.05 and ** *p* < 0.01. (**C**) Expression and localization of TGF-β1 in DRG tissue via immunofluorescence staining. The right image is an enlarged version of the box on the left. Scale bar = 100 μm. (**D**) Expression and localization of TGF-β1 in DRG neuron cells via immunocytochemistry staining. The primary DRG neuron cells were cultured by digesting DRG tissues. Scale bar = 50 μm. n = 3. TGF-β1, transforming growth factor-beta 1. RT-qPCR, real-time quantitative PCR. DRG, dorsal root ganglia.

The L4 and L5 DRG tissues were collected at 1, 4, 7, 14, 21, and 28 days after the transection of the sciatic nerve, and tissue from 0 days was used as a control. RT-qPCR was performed to analyze time-course mRNA expression of TGF-β1 in the DRG. After sciatic nerve injury, the expressions of TGF-β1 gradually increased. At 14 d, expression reached a peak (Figure 1A,B). We also detected the localization of TGF-β1 in L4 and L5 DRG tissues and neuron cells via IF staining. Based on staining results, TGF-β1 is located in cytoplasm and in the nuclei of neuron cells in DRG tissues (Figure 1C) and in primary cultured DRG neuron cells (Figure 1D).

### 3.2. Effects of TGF-β1 on DRG Neuron Cells

**Figure 2:**
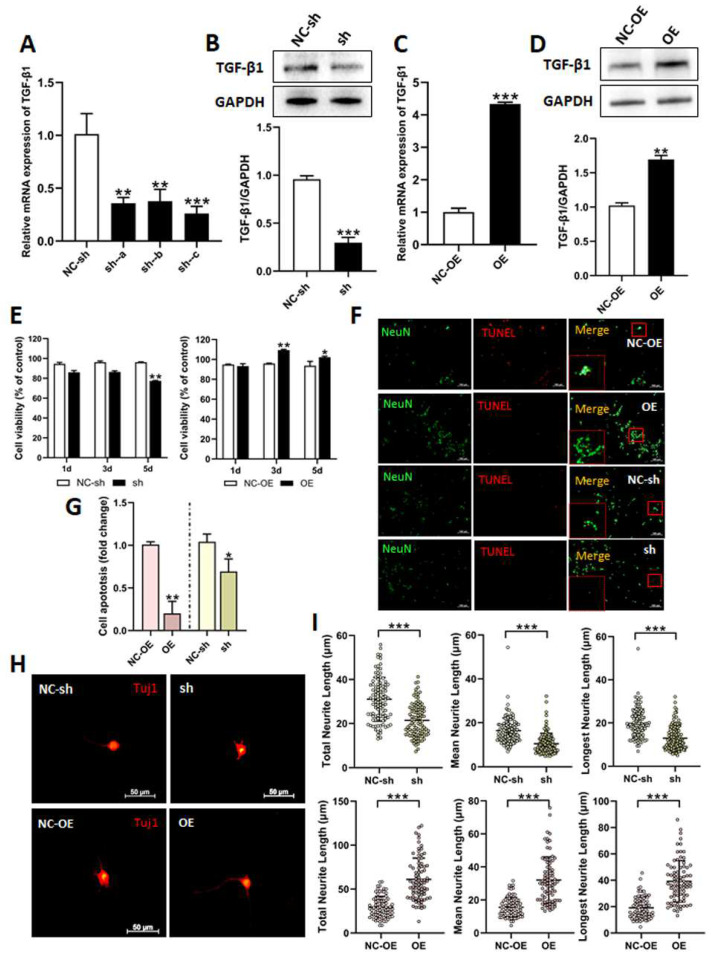
Effects of TGF-β1 on DRG neurons. The shRNA lentivirus (sh-a, sh-b, and sh-c) was prepared for the knockdown of TGF-β1, and lentivirus (OE) was prepared for the overexpression of TGF-β1. (**A**) The expressions of TGF-β1 in DRG neuron cells after transfection with shRNA lentivirus were detected via RT-qPCR. (**B**) The expressions of TGF-β1 in DRG neuron cells after transfection with shRNA lentivirus (sh-c) were detected via Western blot. Versus NC-sh, ** *p* < 0.01 and *** *p* < 0.001. (**C**,**D**) The expressions of TGF-β1 in DRG neuron cells after transfection with overexpression lentivirus were detected via RT-qPCR or Western blot. Versus NC-OE, ** *p* < 0.01 and *** *p* < 0.001. (**E**) Cell viability of DRG neurons after transfection with shRNA lentivirus or overexpression lentivirus prior to 1 d, 3 d, or 5 d. Versus NC-sh or NC-OE, * *p* < 0.05 and ** *p* < 0.01. (**F**) TUNEL staining of DRG neuron cells after transfection with shRNA lentivirus or overexpression lentivirus. Scale bar = 100 μm. (**G**) Statistical analysis of cell apoptosis (**F**). Versus NC-sh or NC-OE, * *p* < 0.05 and ** *p* < 0.01. (**H**) Immunofluorescence staining with Tuj1 of DRG neuron cells after transfection with shRNA lentivirus or overexpression lentivirus. (**I**) Statistical analysis of total neurite length, mean neurite length, and longest neurite length of DRG neurons. *** *p* < 0.001. TGF-β1, transforming growth factor-beta 1. RT-qPCR, real-time quantitative PCR. DRG, dorsal root ganglia. shRNA, small hairpin RNA. NC-sh, shRNA lentivirus negative control. OE, overexpression. NC-OE, overexpression lentivirus negative control. TUNEL, TdT-mediated dUTP Nick-End Labeling.

In order to investigate the biological roles of TGF-β1 in DRG neuron cells, we used lentivirus to knockdown or overexpress TGF-β1. CCK8 assay, TUNEL staining, and IF staining was employed to detect the effects of TGF-β1 on cell viability, apoptosis, and the neurite outgrowth of DRG neurons after the cells had been transfected with lentivirus. Firstly, we used RT-qPCR and Western blot to detect the expressions of TGF-β1 following transfection. The three viruses (sh-a, sh-b, and sh-c) were used to knockdown TGF-β1. The expressions of TGF-β1 were significantly reduced after transfection. However, the expression was lowest when using sh-c (Figure 2A). Therefore, this virus (sh-c) was employed in the following research. The expression of TGF-β1 was also significantly decreased after transfection with sh-c, as detected via Western blot (Figure 2B). The virus (OE) was used to overexpress TGF-β1. The expressions were markedly increased after transfection with OE (Figure 2C,D).

DRG neuron cells were transfected with sh-c or OE and, after 1, 3, and 5 days, the CCK8 assay was applied to detect cell viability. Cell viability had obviously decreased after knockdown at 5 days, but had distinctly increased after overexpression at 3 and 5 days (Figure 2E).

The TUNEL assay was applied to analyze the effect of TGF-β1 on the apoptosis of DRG neuron cells. Based on the statistical results of apoptosis, the apoptosis rate was dramatically decreased after the overexpression of TGF-β1. On the contrary, knockdown of TGF-β1 obviously promoted apoptosis (Figure 2F,G).

Tuj1 is expressed in neurons of nervous systems and has been used widely as a marker of neural differentiation [26]. So, Tuj1 IF staining was adapted to detect the effect of TGF-β1 on the neurite outgrowth of DRG neuron cells. We performed statistical analysis for total neurite length, mean neurite length, and longest neurite length of primary DRG neuron cells after transfection. As shown in the results, the total neurite length, mean neurite length, and longest neurite length were all dramatically raised in the overexpression group compared to the negative control group. However, the neurite lengths were all reduced in the group with a knockdown of TGF-β1 (Figure 2H,I).

**Figure 3:**
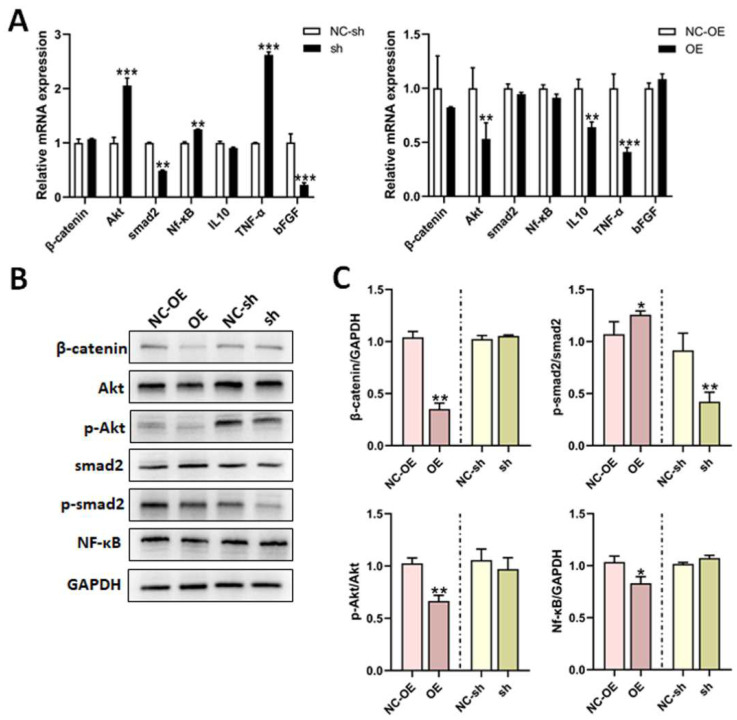
Effects of TGF-β1 on cell factors in DRG neurons. (**A**) The mRNA expressions of β-catenin, Akt, smad2, Nf-κB, IL-10, TNF-α, and bFGF in DRG neuron cells after transfection with shRNA lentivirus or overexpression lentivirus were detected via RT-qPCR. (**B**) The expressions of β-catenin, Akt, p-Akt, smad2, p-smad2, and Nf-κB in DRG neuron cells after transfection with shRNA lentivirus or overexpression lentivirus were detected via Western blot. (**C**) Statistical analysis of (**B**). Versus NC-sh or NC-OE, * *p* < 0.05, ** *p* < 0.01 and *** *p* < 0.001. TGF-β1, transforming growth factor-beta 1. RT-qPCR, real-time quantitative PCR. DRG, dorsal root ganglia. shRNA, small hairpin RNA. NC-sh, shRNA lentivirus negative control. OE, overexpression. NC-OE, overexpression lentivirus negative control. Nf-κB, nuclear factor kappa-B. IL, interleukin. TNF-α, tumor necrosis factor-α. bFGF, basic fibroblast growth factor. GAPDH, glyceraldehyde-3-phosphate dehydrogenase.

Numerous proteins are involved in the neurite outgrowth and apoptosis of DRG neuron cells. We performed RT-qPCR and Western blot to investigate the effects of TGF-β1 on related factors in these processes. The mRNA expressions of Akt, IL-10, and TNF-α were all apparently decreased in DRG neuron cells treated with an overexpression of TGF-β1. However, the mRNA expressions of Akt, Nf-κB, and TNF-α were all sensibly increased, as well as the mRNA expressions of smad2 and bFGF, which were both decreased after the knockdown of TGF-β1 (Figure 3A). Similarly, β-catenin, p-Akt/Akt, and Nf-κB all decreased in the overexpression group, according to Western blot. Additionally, p-smad2/smad2 was significantly reduced in the knockdown group (Figure 3B).

### 3.3. Effects of TGF-β1 on Functional Recovery After Sciatic Nerve Injury

**Figure 4:**
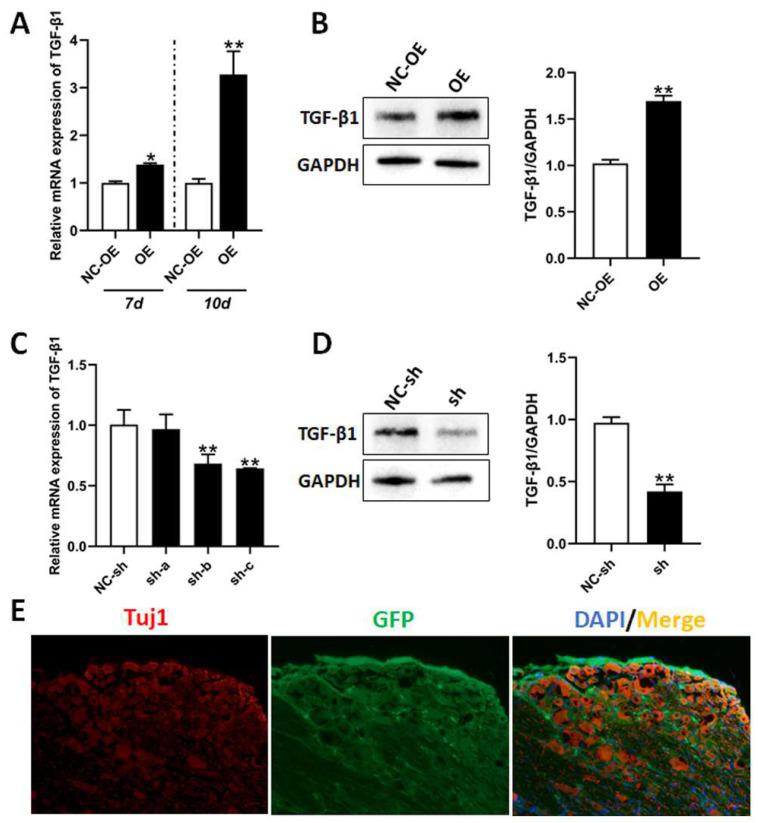
Expressions of TGF-β1 in in vivo model after lentivirus injection. After the rat sciatic nerve regeneration model was established, the lentivirus was intrathecally injected into the L4-L5 foramina. (**A**) The mRNA expressions of TGF-β1 in the DRG at 7 days or 10 days after injection with overexpression lentivirus were detected via RT-qPCR. (**B**) The expressions of TGF-β1 in the DRG at 10 days after injection with overexpression lentivirus were detected via Western blot. (**C**) The mRNA expressions of TGF-β1 in the DRG at 3 days after injection with shRNA lentivirus were detected via RT-qPCR. (**D**) The expressions of TGF-β1 in the DRG at 3 days after injection with shRNA lentivirus (sh-c) were detected via Western blot. (**E**) Immunofluorescence staining with Tuj1 in the DRG after transfection with shRNA lentivirus. The recombination virus vector included the GFP gene. Scale bar = 50 μm. Versus NC-sh or NC-OE, * *p* < 0.05, and ** *p* < 0.01. n = 3. TGF-β1, transforming growth factor-beta 1. RT-qPCR, real-time quantitative PCR. DRG, dorsal root ganglia. shRNA, small hairpin RNA. NC-sh, shRNA lentivirus negative control. OE, overexpression. NC-OE, overexpression lentivirus negative control. GFP, green fluorescent protein.

In order to investigate the roles of TGF-β1 in nerve regeneration and functional recovery after sciatic nerve injury, we performed intrathecal injection with lentivirus to knockdown or overexpress TGF-β1 in sciatic nerve regeneration models. Firstly, RT-qPCR and Western blot was applied to detect expression of TGF-β1 in the DRG after virus injection. The mRNA expression of TGF-β1 was upregulated at 7 days after the virus (OE) had been injected. Surprisingly, the mRNA expression of TGF-β1 was more upregulated in the DRG 10 days after injection (Figure 4A). Meanwhile, the expression of TGF-β1 was also significantly upregulated, as determined via Western blot (Figure 4B). At 3 days after injection with three viruses (sh-a, sh-b, and sh-c), mRNA expressions of TGF-β1 in the DRG were downregulated in both the sh-b and sh-c groups, determined via RT-qPCR (Figure 4C). Similarly, expression of TGF-β1 was significantly downregulated in the DRG injected with sh-c, determined via Western blot (Figure 4D). Due to the green fluorescence protein (GFP) gene being included in the recombination virus vector, the signal of this GFP could be clearly observed in DRG tissues after injection with sh-c (Figure 4E).

**Figure 5:**
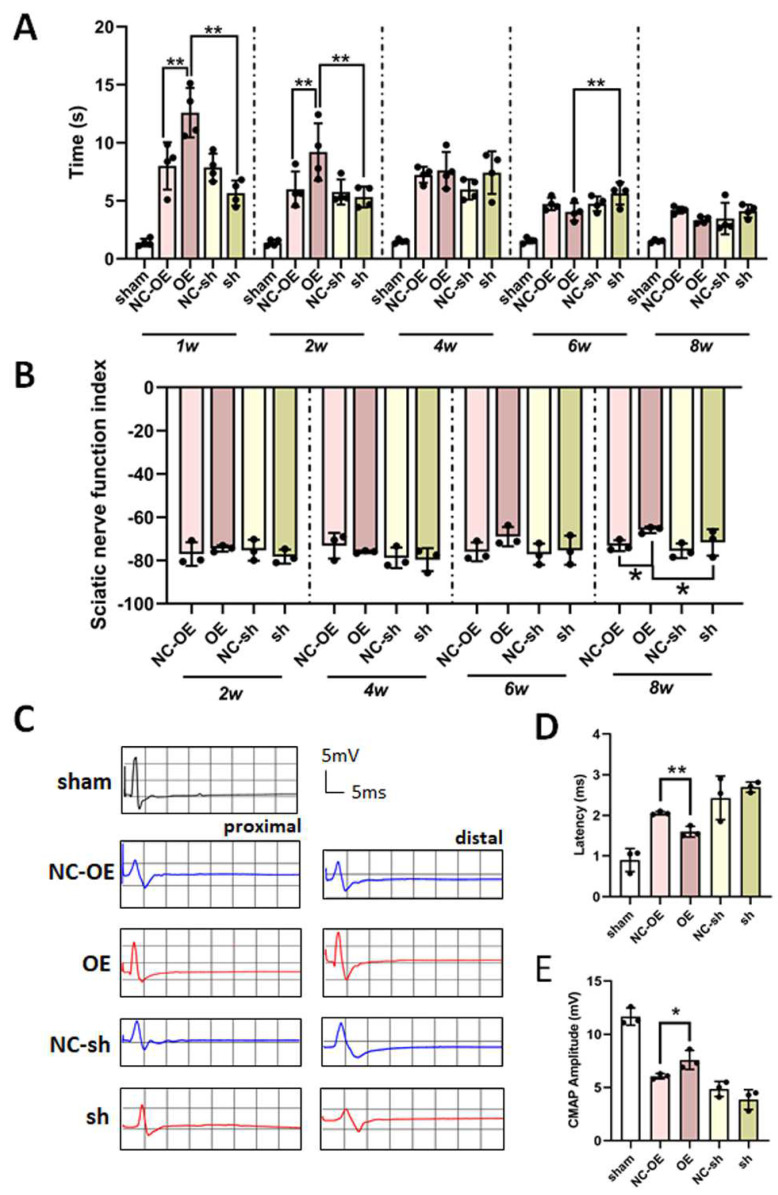
Effects of TGF-β1 on functional recovery after sciatic nerve injury. After the rat sciatic nerve regeneration model was established, the lentivirus was intrathecally injected into the L4-L5 foramina. The thermal pain and sciatic nerve function index were measured at 1, 2, 4, 6, and 8 weeks after lentivirus injection. (**A**) The statistical analysis of withdrawal reflex latency. (**B**) The statistical analysis of sciatic nerve functional index. The compound muscle action potentials (CMAPs) were recorded at 8 weeks after virus injection. (**C**) The representative images of CMAPs. The statistical analysis of latency (**D**) and CMAP amplitude (**E**). * *p* < 0.05, and ** *p* < 0.01. n = 3. TGF-β1, transforming growth factor-beta 1. shRNA, small hairpin RNA. NC-sh, shRNA lentivirus negative control. OE, overexpression. NC-OE, overexpression lentivirus negative control.

Behavioral tests (Catwalk gait analysis and thermal analysis) were applied to measure the functional recovery in sciatic nerve regeneration models. Statistical analysis of the time of contraction, induced by light stimulation, revealed that the time was significantly elongated in the overexpression group at 1 week and 2 weeks after virus injection. However, the time in the knockdown group was shortened (Figure 5A).

At 8 weeks post-virus injection, the SFI scores for TGF-β1 in the overexpression group had obviously ascended compared to those of the negative control group and the knockdown group (Figure 5B). This indicates that TGF-β1 overexpression accelerated the recovery of locomotive function.

An electrophysiological test is recognized as direct evidence reflecting functional recovery [27]. The electrophysiological data show that latency was significantly decreased, and CMAP amplitude was obviously increased in the overexpression group compared to in the negative control group and the knockdown group (Figure 5C,D). The data also indicate that TGF-β1 overexpression improved nerve fibers, innervating the target muscle, and thereby accelerating the functional recovery.

### 3.4. Effects of TGF-β1 on Nerve Regeneration After Sciatic Nerve Injury

**Figure 6:**
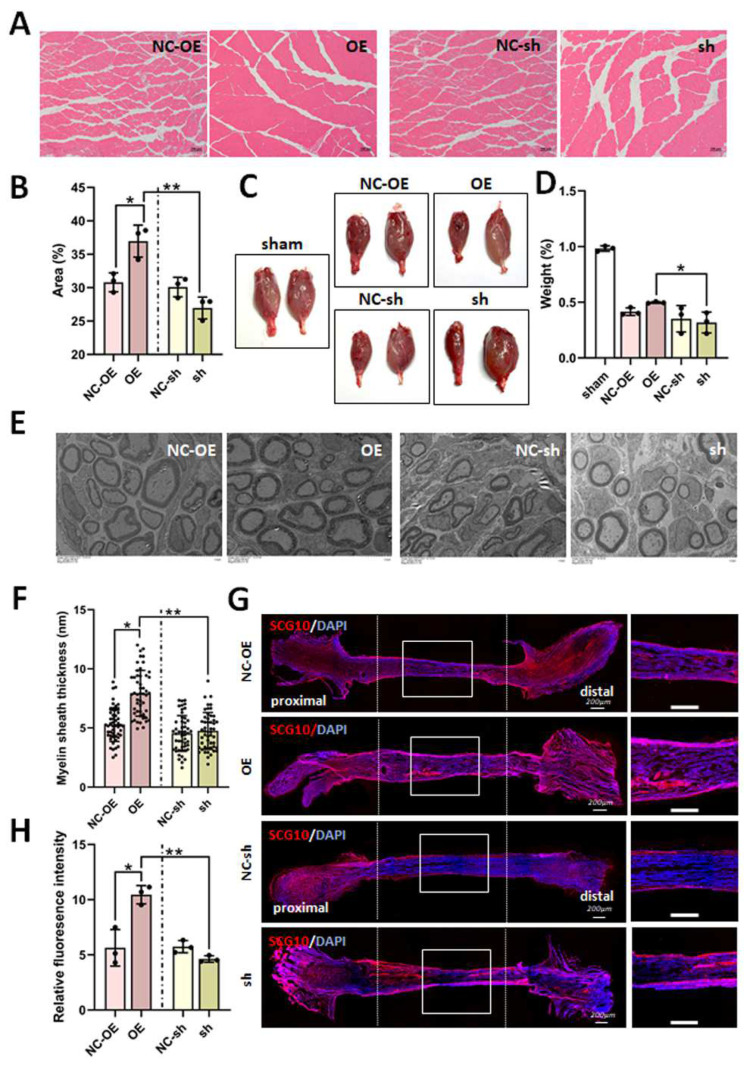
Effects of TGF-β1 on nerve regeneration after sciatic nerve injury. After the rat sciatic nerve regeneration model was established, the lentivirus was intrathecally injected into the L4-L5 foramina. The H&E staining of gastrocnemius muscles, immunofluorescence staining of SCG10, and transmission electron microscope examination of sciatic nerve were performed at 8 weeks after virus injection. (**A**) Representative images of H&E staining. (**B**) Statistical analysis of cross-sectional area of muscle fibers. (**C**) Representative images of gastrocnemius muscles. The left gastrocnemius muscles were from operated limbs, the right muscles were from contralateral limbs. (**D**) Statistical analysis of wet weight ratio of gastrocnemius muscles. (**E**) Representative images of transmission electron microscopy. Scale bar = 5 μm. (**F**) Statistical analysis of myelin sheath thickness. (**G**) Immunofluorescence staining of SCG10 of sciatic nerve. Scale bar = 200 μm. (**H**) Statistical analysis of relative fluorescence intensity. * *p* < 0.05 and ** *p* < 0.01. n = 3. H&E, Haematoxylin and Eosin. TGF-β1, transforming growth factor-beta 1. shRNA, small hairpin RNA. NC-sh, shRNA lentivirus negative control. OE, overexpression. NC-OE, overexpression lentivirus negative control.

Firstly, we investigated the effects of TGF-β1 on nerve regeneration by measuring cross-sectional area of fibers and the wet weight ratio of gastrocnemius muscles, which is the target muscle reinnervation. The cross-sectional area of muscle fibers was obviously enlarged in the overexpression group compared to in the negative control and knockdown groups (Figure 6A,B). Similarly, the wet weight ratio was also increased in the overexpression group (Figure 6C).

Secondly, we observed regenerated myelinated nerve fibers with transmission electron microscopy. The average thickness of the regenerated myelin sheath was distinctly amplified in the overexpression group compared to the negative control and knockdown groups (Figure 6E,F).

The neuron-specific protein SCG10 is also known as stathmin-2 or STMN2, and is a well-known regulator of microtubule dynamics in neurons [28]. Finally, we detected regenerated sciatic nerve fibers via the IF staining of SCG10. A positive signal from SCG10 could be clearly observed in the overexpression group (Figure 6G). So, according to statistical analysis, the relative fluorescence intensity of SCG10 was visibly increased in the overexpression group compared to in the negative control and knockdown groups (Figure 6H). These data indicate that TGF-β1 improves nerve regeneration after sciatic nerve injury.

### 3.5. Mechanisms of TGF-β1 in Nerve Regeneration After Sciatic Nerve Injury

**Figure 7:**
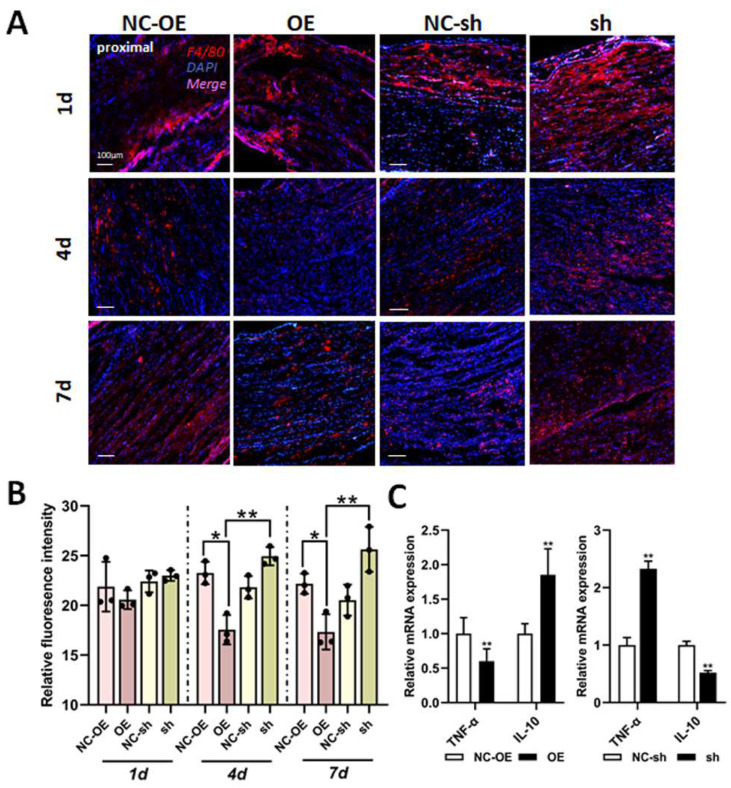
Effects of TGF-β1 on inflammation after sciatic nerve injury. (**A**) The immunofluorescence staining of F4/80 in the sciatic nerve was performed 1, 4, and 7 days after lentivirus injection. (**B**) Statistical analysis of relative fluorescence intensity. (**C**) The mRNA expressions of TNF-α and IL-10 in the sciatic nerve were detected via RT-qPCR. Versus NC-sh or NC-OE, * *p* < 0.05, and ** *p* < 0.01. n = 3. TGF-β1, transforming growth factor-beta 1. RT-qPCR, real-time quantitative PCR. shRNA, small hairpin RNA. NC-sh, shRNA lentivirus negative control. OE, overexpression. NC-OE, overexpression lentivirus negative control. IL, interleukin. TNF-α, tumor necrosis factor-α.

Inflammatory response is a physiological response of the body to harmful stimuli. Recently, it has become gradually accepted by most scholars that neuroinflammation can be extended to the inflammatory response of the PNS, especially that of the sciatic nerve and DRG [29]. Furthermore, immune cells, cytokines, and chemokines are the main mediators of the inflammatory response. Therefore, we tried to investigate the effects of TGF-β1 on infiltrated macrophages and cytokines (TNF-α and IL-10) in the sciatic nerve via IF staining and RT-qPCR. After anti-F4/80 IF staining, a positive signal was clearly reduced in the proximal end tissues of the overexpression group (Figure 7A,B). The mRNA expressions of pro-inflammatory factor (TNF-α) dramatically decreased; however, expressions of the anti-inflammatory factor (IL-10) were significantly increased in the overexpression group (Figure 7C). These data indicate that TGF-β1 inhibits infiltrated macrophages and pro-inflammatory factor expression and promotes anti-inflammatory factor expression during nerve regeneration.

**Figure 8:**
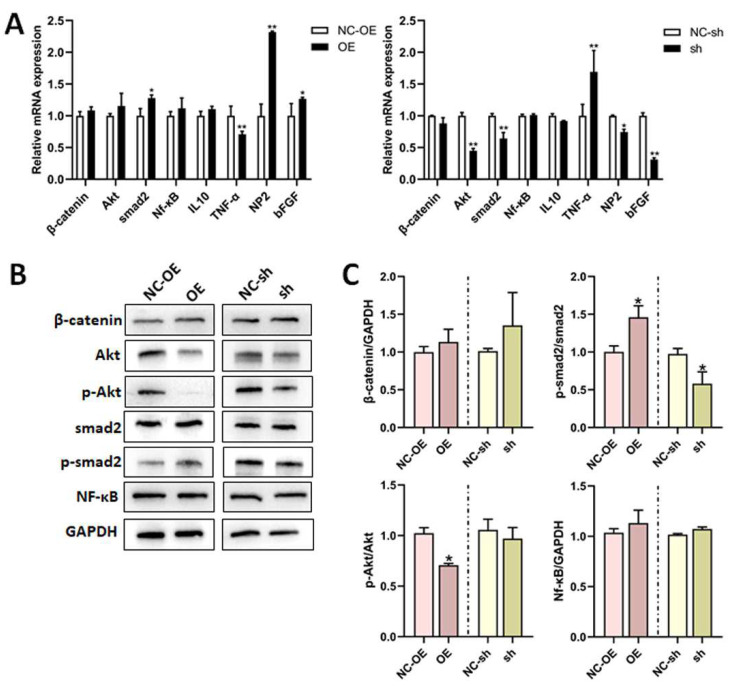
Effects of TGF-β1 on cell factors after sciatic nerve injury. After the rat model of sciatic nerve regeneration was established, the lentivirus was intrathecally injected into the L4-L5 foramina. (**A**) The mRNA expressions of β-catenin, Akt, smad2, Nf-κB, IL-10, TNF-α, NP2, and bFGF in the DRG after lentivirus injection were detected via RT-qPCR. (**B**) The expressions of β-catenin, Akt, p-Akt, smad2, p-smad2, and Nf-κB in the DRG after lentivirus injection were detected via Western blot. (**C**) Statistical analysis of (**B**). Versus NC-sh or NC-OE, * *p* < 0.05, and ** *p* < 0.01. n = 3. TGF-β1, transforming growth factor-beta 1. RT-qPCR, real-time quantitative PCR. DRG, dorsal root ganglia. shRNA, small hairpin RNA. NC-sh, shRNA lentivirus negative control. OE, overexpression. NC-OE, overexpression lentivirus negative control. Nf-κB, nuclear factor kappa-B. IL, interleukin. TNF-α, tumor necrosis factor-α. NP2, neuronal pentraxin 2. bFGF, basic fibroblast growth factor. GAPDH, glyceraldehyde-3-phosphate dehydrogenase.

We also performed RT-qPCR and Western blot to investigate the effects of TGF-β1 on related factors and signal pathways in DRG tissues. The mRNA expressions of NP2, smad2, and bFGF were all remarkably increased; However, TNF-α expression was decreased in the overexpression group (Figure 8A). Conversely, the mRNA expressions of NP2, Akt, smad2, and bFGF were all remarkably decreased, and TNF-α expression was increased in the knockdown group (Figure 8A). The p-smad2/smad2 expression was clearly upregulated; However, p-Akt/Akt was downregulated in the overexpression group, as determined via Western blot. Additionally, p-smad2/smad2 was obviously downregulated in the knockdown group (Figure 8B,C).

## 4. Discussion

The repair and regeneration of peripheral nerves after injury is still an important research field in modern neurobiology. It is common sense that WD will occur at the distal end of the injured nerve after peripheral nerve injury [30]. On the one hand, it is quite precisely regulated and a large number of cell signals and growth factors, such as SCs and macrophages, are involved in this process [31,32]. On the other hand, the injury signal is retrogradely transported to the neuronal cell body by a calcium wave to cause gene reprogramming of the neurons after the decomposition of the myelin sheath, activate the neurons’ regeneration ability, and then repair the damaged nerve from the proximal and distal ends [33,34]. So, WD is prepared for future nerve regeneration. In our previous study, we performed gene chip microarrays to analyze gene expression files in early WD after sciatic nerve injury [19,35]. It was assumed that TGF-β1 played a crucial role in WD. In this study, it is our aim to explore the biological role and possible mechanism of TGF-β1 in the DRG in the process of repair and regeneration after peripheral nerve injury.

First of all, we detected the time-course expression and localization of TGF-β1 in the DRG after the rat sciatic nerve transection. Subsequently, we investigated the effects of TGF-β1 on cell viability, neurite outgrowth, and apoptosis of primary cultured DRG neuron cells with an in vitro model. Afterwards, we further investigated the effects of TGF-β1 on nerve regeneration and neurological function recovery, as well as its possible mechanism, through an in vivo model. The data from the time-course expression in the DRG tissues indicate that TGF-β1 expressions gradually increased and reached a peak at 14 d after sciatic nerve transection. Additionally, the expressions of TGF-β1 were mainly located in the neuron cells in the DRG. Significant changes in TGF-β1 expression after nerve injury are a hint that TGF-β1 may play an important role in the DRG.

The neurite outgrowth of neurons represents a key segment of neural development and regeneration [36,37]. It has been reported that TGF-β1 promotes the re-elongation of injured axons. In this study, lentivirus was used to knockdown or overexpress TGF-β1, and primary cultured DRG neuron cells were subjected to IF staining to determine the beneficial effects of TGF-β1 on neurite outgrowth. It was exciting that TGF-β1 improved the cell viability, promoted neurite outgrowth, and restrained apoptosis in DRG neuron cells. Numerous proteins and signal pathways are involved in the neurite outgrowth and apoptosis of DRG neuron cells [38]. In the primary DRG neuron cells, it is shown that TGF-β1 visibly increases the phosphorylation of smad2. These data may be a demonstration that TGF-β1 plays a critical role in neurite outgrowth by promoting phosphorylation of smad2. It is also indirect evidence for the interrelationship between TGF-β1 and injured neurons.

In this study, we further investigated the roles of TGF-β1 in nerve regeneration and functional recovery with in vivo models. The intrathecal injection with lentivirus was adapted to trigger a knockdown or overexpression of TGF-β1 in L4-L5 DRG. Peripheral nerve injury often leads to temporary or permanent neurological dysfunction, mainly manifesting as motor or/and sensory dysfunction or neurotrophic changes [39,40]. Walking track analysis and thermal pain analysis are used to analyze the spontaneous recovery of sensory and motor function, and their values also reflect the degree of nerve dysfunction. The data of thermal pain analysis indicate that TGF-β1 could alleviated neuropathic pain when compared to a negative control in an early stage (1 and 2 weeks) of sciatic nerve injury. Additionally, the data of SFI could also indicate that TGF-β1 can improve the recovery of locomotive function when compared with a negative control at a late stage (8 weeks) of injury. The electrophysiological test is recognized as direct evidence reflecting functional recovery [27]. These data also indicate that TGF-β1 improves nerve fibers, innervating target muscle and thereby accelerating neuronal function recovery.

After peripheral nerve injury, if the cell body of neurons does not die, the nerve can recover to a certain extent. This recovery is mainly represented by the regeneration of nerve axons [41]. The basis of nerve regeneration is the regeneration of nerve axons, which can cross the gap to the target [42,43]. In this study, we constructed a sciatic nerve regeneration model with a silicone tube sutured between two stump ends to investigate the neuroprotective role of TGF-β1. SCG10, a microtubule dynamic regulator, was located in the membrane of the axon and the growth cone [28]. The positive signals could be clearly observed, and relative fluorescence was visibly increased in the TGF-β1 overexpression group compared to the negative control. This is direct evidence that TGF-β1 improves the regeneration of nerve axons.

Peripheral nerve injury can lead to changes in target organs, including skeletal muscle atrophy and sensory degeneration [44,45]. All voluntary activities in daily life are achieved through muscle contraction. Once the connection between nerve and target muscle is broken, the target muscle loses its source of nutrition due to the loss of nerve innervation, and this can lead to skeletal muscle atrophy [46]. So, we used wet weight ratio and a cross-sectional area of muscle fibers to evaluate the effects of TGF-β1 on nerve regeneration. As shown in the results, TGF-β1 can increase the wet weight ratio and cross-sectional area of muscle fibers. Therefore, TGF-β1 plays a beneficial role in nerve regeneration after injury.

Peripheral nerve injury can lead to the myelin degeneration of distal axons [33,39]. After WD, the whole axonal structure becomes degenerated, and damaged cells are phagocytosed by macrophages and SCs to maintain a good regeneration condition for new axons [47]. The reformation of the myelin sheath is an important foundation for the functional recovery of myelinated nerve fibers [48,49]. In this study, we observed regenerated myelinated axons via transmission electron microscopy. TGF-β1 can amplify the average thickness of the regenerated myelin sheath. SCs play a crucial function in the reformation of the myelin sheath. In the future, we will further investigate the regulation role of TGF-β1 on SCs in the reformation process of the myelin sheath.

The influences on inflammation in the PNS are more controversial [50]. On the one hand, the inflammatory response plays a significant role in the process of nerve regeneration [47]. On the other hand, the inflammatory response also leads to neuropathic pain [51]. It has been reported that the early inhibition of the inflammatory response after nerve injury can reduce NPP and accelerate axonal regeneration [52]. The main cells involved in the inflammatory response are macrophages, which have the function of promoting nerve regeneration. In this study, we found that TGF-β1 reduces the infiltrated macrophages and pro-inflammatory factor expression and promotes anti-inflammatory factor expression. So, TGF-β1 facilitated the inhibition of the inflammatory response after injury.

In summary, after a sciatic nerve injury, TGF-β1 in the DRG regulates the viability, apoptosis, and neurite outgrowth of DRG neuron cells, alleviates inflammatory response, relieves neuropathic pain, and improves nerve regeneration and neuronal function recovery via the TGF-β1/smad2 pathway. This study provides an experimental and theoretical basis for the application of TGF-β1 for nerve regeneration and functional recovery after peripheral nerve injury in clinical practice in the future.

## Figures and Tables

**Table 1:** The sequences of primers.

Gene	Primer	Sequence (5′-3′)
GAPDH	Forward	TGGAGTCTACTGGCGTCTT
	Reverse	TGTCATATTTCTCGTGGTTCA
TGF-β1	Forward	GGCTGAACCAAGGAGACGG
	Reverse	CCTCGACGTTTGGGACTGAT
TNF-α	Forward	ATGGGCTCCCTCTCATCAGT
	Reverse	GCTTGGTGGTTTGCTACGAC
IL-10	Forward	GGGAGAGAAGCTGAAGACCC
	Reverse	ACACCTTTGTCTTGGAGCTTATTA
Akt	Forward	CCGGTGAACTCTGACCCTTG
	Reverse	GGCCGCAGCGTCTTCAT
β-catenin	Forward	GGAGCTAAAATGGCAGTGCG
	Reverse	GGCCAGAATGATGAGCTTGC
bFGF	Forward	CCCGCACCCTATCCCTTCACAGC
	Reverse	CACAACGACCAGCCTTCCACCCAAA
NF-κB	Forward	ACAATAACCCCTTTCAAGTTCCC
	Reverse	AATCGGATGCGAGAGGACAG
Smad2	Forward	GCCGCCCGAAGGGTAGAT
	Reverse	AGACCCACCGGCTGATTTTT
NP2	Forward	GAGAAGTCCCTGCTCCACAA
	Reverse	TTGAATGCACTGTTGCCTCTCT

**Table 2:** The antibodies used.

Antibody	Sources	Catalogue Number
GAPDH	Proteintech	60004-1-Ig
TGF-β1	Abcam	Ab215715
smad2	Abcam	Ab40855
p-smad2	Abcam	Ab188334
AKT	Cell signaling	4691T
p-AKT	Cell signaling	4060T
β-catenin	Cell signaling	8480T
NF-kB	Cell signaling	8214T
F4/80	Abcam	Ab307470
TUBB3 (Tuj1)	Abways	AB0043
TUBB3 (Tuj1)	Proteintech	66375-1-Ig
STMN2 (SCG10)	Proteintech	10586-1-AP
NeuN	Abcam	Ab177487
Goat Anti-Mouse IgG HRP	Abways	AB0102
Goat Anti-Rabbit IgG HRP	Abways	AB0101
Cy3-conjugated Goat Anti-Rabbit	Proteintech	SA00009-2
CoraLite488-conjugated Goat Anti-Rabbit	Proteintech	SA00013-2
CoraLite488-conjugated Goat Anti-Mouse	Proteintech	SA00013-1

## Data Availability

All data generated or analyzed during this study are included in this published article.
